# Statement of Retraction: Exosomal long non-coding RNA-LINC00839 promotes lung adenocarcinoma progression by activating NF-κB signaling pathway

**DOI:** 10.1080/07853890.2026.2651016

**Published:** 2026-04-17

We, the Editors and Publisher of *Annals of Medicine*, have retracted the following article:

Sun, Y., Zhen, F., Wang, H., Liang, X., Wang, Y., Wang, F., & Hu, J. (2024). Exosomal long non-coding RNA-LINC00839 promotes lung adenocarcinoma progression by activating NF-κB signaling pathway. *Annals of Medicine*, *56*(1). https://doi.org/10.1080/07853890.2024.2430029

Since publication, concerns were identified by the publisher regarding potential ethical approval discrepancies, image integrity of Western Blots and the qRT-PCR methodology described in the article. When approached for an explanation, the authors responded, however, they were unable to address the concerns raised.

As verifying the validity of published work is core to the integrity of the scholarly record, we are therefore retracting the article. The authors do not agree with the retraction.

We have been informed in our decision-making by our editorial policies and the COPE guidelines.

The retracted article will remain online to maintain the scholarly record, but it will be digitally watermarked on each page as ‘Retracted’.

